# Curcumin-Activated Mesenchymal Stem Cells Derived from Human Umbilical Cord and Their Effects on MPTP-Mouse Model of Parkinson's Disease: A New Biological Therapy for Parkinson's Disease

**DOI:** 10.1155/2020/4636397

**Published:** 2020-02-17

**Authors:** Yun-Liang Wang, Xin-Shan Liu, Shan-Shan Wang, Peng Xue, Zhi-Lei Zeng, Xiao-Peng Yang, Si-Miao Zhang, Wei Zheng, Linlin Hua, Jin-Feng Li, Hai-Tao Wang, Shang Guo

**Affiliations:** ^1^Department of Neurology, The Second Affiliated Hospital of Zhengzhou University, 2 Jingba Road, Zhengzhou 450014, China; ^2^Department of Neurology, The 960th Hospital, 20 Zhanbei Road, Zibo 255300, China; ^3^Sanbo Brain Hospital, Capital Medical University, 50 Xiang Shan Yikesong Road, Beijing 100093, China; ^4^Department of Oncology, Chinese PLA General Hospital, Beijing 100037, China; ^5^Department of Cardiology, The 970th Hospital, No. 7 Zhichunan Road, Shandong 264002, China; ^6^Shanghai Jiao Tong University Affiliated Sixth People's Hospital, Shanghai 200233, China

## Abstract

**Background:**

The aim of this study was to investigate the effects of human umbilical cord mesenchymal stem cell activated by curcumin (hUC-MSCs-CUR) on Parkinson's disease (PD). hUC-MSCs can differentiate into many types of adult tissue cells including dopaminergic (DA) neurons. CUR could protect DA neurons from apoptosis induced by 6-hydroxydopamine (6-OHDA). Therefore, we used the hUC-MSCs activated by CUR for the treatment of PD in an animal model.

**Methods:**

The hUC-MSCs-CUR was transplanted into the MPTP-induced PD mouse models via the tail vein. We found that hUC-MSCs-CUR significantly improved the motor ability, increased the tyrosine hydroxylase (TH), dopamine (DA), and Bcl-2 levels, and reduced nitric oxide synthase, Bax, and cleaved caspase 3 expression in PD mice. The supernatant of hUC-MSCs-CUR (CM-CUR) was used to stimulate the SH-SY5Y cellular model of PD; cell proliferation, differentiation, TH, and neuronal-specific marker microtubular-associated protein 2 (MAP2) expressions were examined.

**Results:**

Our data showed that CM-CUR significantly promoted cell proliferation and gradually increased TH and MAP2 expression in SH-SY5Y PD cells. The beneficial effects could be associated with significant increase of rough endoplasmic reticulum in the hUC-MSCs-CUR, which secretes many cytokines and growth factors beneficial for PD treatment.

**Conclusions:**

Transplantation of hUC-MSCs-CUR could show promise for improving the motor recovery of PD.

## 1. Introduction

Pathological features of Parkinson's disease (PD) include the progressive loss of dopaminergic (DA) neurons selectively in the pars compacta of the substantia nigra (SN) and corpus striatum (CS) and the presence of eosinophilic Lewy bodies in the residual DA neurons. In this condition, the amount of DA in the CS is decreased and patients present clinical symptoms such as tremor, rigidity, and bradykinesia [1–3]. Currently, the available treatments of PD are drugs and surgery [4–6]. Although levodopa can improve the early symptoms of patients with PD, it cannot prevent the progressive reduction of DA neurons at the late stage of PD; moreover, complications such as wearing-off, on-off phenomena, and drug-induced dyskinesia can occur [4]. Surgery improves tremor and rigidity to some extent, but it cannot prevent the progression of PD, and the risks of surgery are relatively high [5, 6].

It is well known that stem cells have the unique characteristics of self-renewal and multipotential differentiation. Under suitable conditions or appropriate induction, they can differentiate into many types of adult tissue cells, including DA neurons [7, 8]. This multipotential differentiation ability provides great hopes for the treatment of neurodegenerative diseases such as PD [9]. At present, available stem cells mainly include neural stem cells (NSCs), embryonic stem cells (ESCs), and mesenchymal stem cells (MSCs) [10–16]. MSCs are multipotent cells capable of differentiating into dopaminergic (DA) neurons, which is one of the major cell types damaged in PD. MSCs include bone marrow mesenchymal stromal cells (BM-MSCs) and human umbilical cord mesenchymal stromal cells (hUC-MSCs) [17–20]. Systemic BM-MSC transplantation can prevent neuronal damage in the MPTP-lesioned rats and is considered as a potential treatment for PD during the early stage of disease development. On the other hand, bone marrow mononuclear cells (BMMCs) accelerated DA damage and induced motor impairment and immobility behavior [17]. A clinical trial showed that MSC administration is feasible in subjects with progressive supranuclear palsy (PSP), a rare, severe, and no-option form of PD [18]. The common methods of MSC transplantation include intravenous injection, intrathecal injection, and local brain injection. In addition, the intranasal (IN) route was also validated as being potentially a safe, easy, and inexpensive alternative route for MSC treatment in neurodegenerative disorders [19]. hUC-MSCs are primitive and possess multiple advantages including ethical acceptance, less invasive procedure for isolation, low immunogenicity, high proliferation capacity, and multilineage differentiation capability [21–23]. Therefore, hUC-MSCs have become an important source of cells for the treatment of diseases. Recent studies demonstrated that activation of hUC-MSCs before transplantation could yield better effects [22, 23]. *In vivo* experiments were performed by inducing hUC-MSCs into TH-positive cells and transplanting them into the CS of PD rats; these cells survived for more than 4 months, and the cells migrated caudally and rostrally by approximately 1.4 mm. More importantly, the behaviors of these rats were improved. Therefore, if hUC-MSCs are appropriately stimulated, they should have abilities that are critical for the treatment of PD.

In the present study, curcumin (CUR) was selected to activate the hUC-MSCs. CUR has wide pharmacological functions such as antioxidation, anti-inflammation, and lowering blood lipids. Studies about the treatment of PD with CUR have already been published [24, 25]. In a PC12 cellular model of PD made using 1-methyl-4-phenylpyridine (MPP+), it was confirmed that CUR protected from MPP+-induced apoptosis [26]. An animal model of PD demonstrated that CUR protected DA neurons from apoptosis induced by 6-hydroxydopamine (6-OHDA) [27, 28]. In 2012, researchers also found that CUR combined with *α*-synuclein (*α*-Syn) prevented accumulation of *α*-Syn in neurons. These results could have significant implications for the treatment of PD [29]. Some researchers pointed out that CUR cannot pass through the blood-brain barrier (BBB), but many studies report that the hydrophobicity of the CUR molecule suggests the possibility of BBB penetration and accumulation in the brain. No matter whether CUR can get through the BBB, it exhibits extremely low bioavailability, mainly due to its poor aqueous solubility, poor stability in solution, and rapid intestinal turnover and hepatic metabolism. hUC-MSCs can pass through BBB and have a strong differentiation potential [21–23].

Therefore, based on the advantages and disadvantages of hUC-MSCs and CUR, the aim of the present study was to examine the effects of hUC-MSCs activated by CUR (hUC-MSCs-CUR) for the treatment of PD, compared with the effects of hUC-MSCs without any treatment.

## 2. Material and Methods

### 2.1. Materials

Umbilical cord tissues were obtained from healthy pregnant women admitted to our hospital for delivery. These women signed a written informed consent. C57BL mice (6-8 weeks) were ordered from Beijing Vital River Laboratory Animal Technology Co. (China). Consumables such as F12 culture medium and pipettes were ordered from Sigma (St. Louis, USA). CUR, 1-methyl-4-phenyl-1,2,3,6-tetrahydropyridine (MPTP), and MPP+ were ordered from Sigma (St. Louis, USA). Fetal calf serum was from HyClone (Waltham, USA). All ELISA kits were from IBL (Germany). All antibodies were purchased from Abcam (UK), BD Biosciences (San Jose, CA), BD (Franklin Lakes, NJ), and BioLegend (Cambridge, UK). Chromogenic kits were ordered from Shanghai to Ruicheng Biotechnology Co. Ltd (Shanghai, China). The SH-SY5Y cell line was obtained from the Shanghai Institute of Cell Biology of the Chinese Academy of Sciences.

### 2.2. Ethical Statement

All the animal experiments described in this study and umbilical cord tissue collection were approved by the Ethics Committee of the 148th Hospital and the Second Affiliated Hospital of Zhengzhou University.

### 2.3. Isolation and Identification of hUC-MSCs

hUC-MSCs were isolated from human umbilical cords and cultured as previously described [22]. Cultured cells were phenotypically characterized for surface antigen expression by fluorescence-activated cell sorting. Cells from passage 3 were harvested by 0.05% trypsin/EDTA, resuspended in phosphate-buffered saline, and counted. Then, 1.0 × 10^5^ cells were stained for CD19 and CD34 (BD Biosciences, San Jose, CA), CD11b and CD73 (BD Immunocytometry Systems, Franklin Lakes, NJ), and CD90, CD105, CD45, and HLA-DR (BioLegend, Cambridge, UK). Samples were analyzed with a LSR II flow cytometer (BD Biosciences), and at least 10,000 events were acquired for each sample. Data acquisition and analysis were performed using the FACS DIVA software (BD Biosciences).

### 2.4. Adipogenic Differentiation

hUC-MSCs at passage 3 were seeded in six-well plates in complete medium in triplicate at a final density of 5000 cells per cm^2^ according to the previous report with some modifications [30] Forty-eight hours later, designated as day 0, differentiation was initiated using the adipogenic induction medium (AdvanceSTEM adipogenic differentiation medium supplemented with 10% AdvanceSTEM stem cell growth supplement, Thermo Scientific, Rockford, IL), according to the manufacturer's instructions. The medium was changed every 3-4 days, and the experiment was terminated after 3 weeks. The differentiated hUC-MSCs were fixed with 4% paraformaldehyde (PFA) and stained with oil-red-O (IHC World, Woodstock, MD) to visualize cytoplasmic lipid-rich vacuoles.

### 2.5. Osteogenic Differentiation

Osteogenic differentiation was induced by treating subconfluent MSC cultures at passage 2 with osteogenic induction medium (AdvanceSTEM osteogenic differentiation medium, supplemented with 10% AdvanceSTEM stem cell growth supplement, Thermo Scientific) for 21 days, according to the manufacturer's instructions and previous report [30]. Experiments were performed in triplicate. The osteogenic potential was examined for extracellular matrix calcification by von Kossa's method using a commercially available kit (IHC World). Cultures were treated with silver nitrate for 60 min at room temperature under ultraviolet light, followed by treatment with sodium thiosulphate for 5 min. The cells were counterstained with nuclear fast red and then photographed using a phase contrast microscope. The ImageJ software was used for the quantification of the mineralized matrix.

### 2.6. Chondrogenic Differentiation

Chondrogenesis was induced in micromass pellet cultures according to the previous report with some modifications [30]. Micromass pellet cultures were prepared from 1.0 − 2.5 × 10^5^ hUC-MSCs (passage 3) in 15 mL polypropylene tubes that were centrifuged at 150 g for 10 min in complete medium. Cell pellets were incubated with induction medium (AdvanceSTEM chondrogenic differentiation medium, Thermo Scientific) for 3 weeks. Each pellet was paraffin-embedded after dehydration and cut into thin sections (4-5 *μ*m). The sections were analyzed for chondrogenic differentiation using a commercially available Alcian blue kit (IHC World, Woodstock). For staining, sections were fixed with 4% PFA and washed with distilled water followed by treatment with Alcian blue for 20 min. After 21 days, the micromass cultures were fixed with methanol and stained with Alcian blue. Alcian blue was extracted with 6 M guanidine HCl ,and absorbance was read at 620 nm. All experiments were performed in triplicate.

### 2.7. Preparation of hUC-MSCs-CUR

The hUC-MSCs were activated using different concentrations of CUR (0, 1, 2.5, 5, 10, 15, 20, and 25 *μ*mol/L). Cell proliferation was detected using a CCK-8 assay kit (each group in triplicate, performed three times). A DMSO solution with the same concentration as in the CUR groups was used as control to detect whether DMSO had a toxic effect on the cells.

### 2.8. Preparation of Animal Model of PD

Female C57BL mice (6-8 weeks) weighing approximately 20 ± 5 grams were used for the preparation of the animal models of PD, as described previously [31]. Twenty mice were randomly selected as controls. The remaining 80 mice were injected intraperitoneally with 250 mg/kg of probenecid and with MPTP (subcutaneous, 25 mg/kg) 30 min later and twice a week for 5 weeks. After three injections, mice presented with symptoms such as severe tremors, unwillingness to move, uncoordinated gait, erected tail, hair shedding, and occasional seizure-like attacks. Nevertheless, 2 h after injection, these reactions slowly recovered to normal, but with the increasing number of MPTP injection, the symptom duration gradually became longer and the symptoms of PD gradually became stable. Five weeks later, the open field test (Beijing Jinyuan Kechuang Technology Co. Ltd, China) and rotarod test (Ugo Basile, USA) were used to verify whether mouse models of PD were successfully established.

### 2.9. Open Field Test

A 30 × 30 × 15 cm glass plastic chamber was made, with 6 × 6 cm grids at the bottom of the box. The test was performed in a quiet and dim environment. After mice had adapted to the environment for 10 min, the number of rearing and ambulation was calculated within 5 min. Five measurements were made, and the mean value was used for analysis.

### 2.10. Rotarod Test

Mice were pretrained for 3 days on a rotarod. Mice with uncoordinated movement were excluded. The mice were placed on the rotating rotarod (16 rpm), and time was counted. Latency to fall was defined as the time of the mice staying on the rotarod, that is, time for the mice falling for the first time. The number of times falling from the rod within 2 min (drop frequency) represented the ability of movement and coordination. Every mouse was evaluated thrice at an interval of 30 min. The mean value was used for analysis. According to the literature [22, 32], in the open field test, the number of rearing was less than 15 times/5 min and ambulation was less than 55 times/5 min. In the rotarod test, a successful establishment of the mouse model of PD was verified as less than 30 s of latency and more than 20 drops/2 min. Five weeks later, after accidentally dead mice were excluded, the total number of mice that met the criteria stated as above was 60.

These mice were randomly assigned to four groups: (1) model group (mice with MPTP and probenecid without any treatment), (2) MSC group (injected with hUC-MSC suspension through the tail vein), (3) MSC-CUR group (injected with hUC-MSCs-CUR suspension through the tail vein), and (4) F12 group (injected with an equal amount of F12 culture medium). Before injection, the cell suspension was washed with PBS three times to remove serum and resuspended in 200 *μ*L of F12 medium (passage 4, approximately 10^7^/cells/kg). hUC-MSCs and hUC-MSCs-CUR suspension were injected into PD mice once a week for 8 weeks. In order for it to fully show the effectiveness of MSC function, the mice were not treated with any material in the next 8 weeks after MSC injection. At the end of 8 weeks, the mice were tested with the open field test and rotarod test again and sacrificed for rapid dissection of whole brains, which were fixed in 4% PFA.

### 2.11. Expression of TH in the SN and CS

Immunohistochemistry was used to detect the expression of TH in the SN and CS. Paraffin sections (4 *μ*m) were deparaffinized until dehydration and then incubated in 3% H_2_O_2_ for 10 min. Sections were washed in distilled water, soaked in phosphate-buffered saline (PBS) for 5 min, and then incubated with rabbit anti-TH antibody solution (1 : 300, Abcam, Cambridge, UK) at 4°C overnight. HRP-conjugated secondary antibodies were used to visualize the staining (1 : 2000, Golden Bridge International, Beijing, China). Ten serial CS sections (at an interval of 50 *μ*m for each section) from each animal (*n* = 5 for each group) were used to quantify the expression of TH. The staining was analyzed with the Image-Pro Plus 5.0 software (Media Cybernetics, USA).

### 2.12. Expression of TH, NADPH-d, Bcl-2, Bax, and Cleaved Caspase 3

The CS from one hemisphere was isolated from PD mice of each group after the behavior test (5 mice per group). The CS samples were homogenized in ice-cold RIPA lysis buffer (Beyotime, Shanghai, China). The homogenized samples were centrifuged at 12,000 g for 20 minutes at 4°C. The supernatant was collected for western blot. The proteins were separated by SDS-PAGE electrophoresis and transferred to polyvinylidene difluoride (PVDF) membranes (R&D System, Minneapolis, MN, USA). The membranes were blocked with 5% nonfat dry milk in Tris-buffered saline (TBS) with Tween (TBST; TBS plus 0.1% Tween 20) for 1 h and incubated with primary antibodies overnight at 4°C. The following primary antibodies were used: TH, NADPH-d, Bcl-2, Bax, and cleaved caspase 3 (rabbit IgG, 1 : 1000, Abcam, Cambridgeshire, UK). The following secondary antibodies were used: goat anti-rabbit IgG/HRP (1 : 5,000, Golden Bridge International, Beijing, China) and rabbit anti-goat IgG/HRP (1 : 5,000, Golden Bridge International, Beijing, China). The intensity of the bands was quantified using the ImageJ software. The level of expression for the target protein was calculated as the ratio of the band intensity of the target protein over that of *β*-actin.

### 2.13. Detection of DA Levels in the CS of PD Mice

The CS from five mice were stored in liquid nitrogen. DA was measured as described previously [33]. Briefly, samples were thawed to 0°C and distributed into new 1.5 mL microcentrifuge tubes (125 *μ*L/tube); 500 *μ*L of acetonitrile (An)/0.4% glacial acetic acid (HAc) at -20°C was added to each tube. The tubes were vortexed for 20 s and centrifuged for 15 min at 12,000 g at -2°C. Supernatant evaporated under vacuum in a CentriVap™ Concentrator (Labconco). The dry remains were dissolved in 100 *μ*L of mobile phase A and placed in autosampler vials. Quantification of DA was performed using the PowerChrom software (eDAQ Pty Ltd., Deniston East, Australia) with standard curves and the peak area method. ISO was used as an internal standard to compensate for variations in the recovery rate during the purification process. HPLC-ECD was performed in triplicate.

### 2.14. MSC Tracking

MSCs were labeled with DiI (Sigma, St. Louis, USA) and administered through the tail vein to the MSC group and MSC-CUR group mice. One day later, the mice were perfused intracardially with PBS followed by 4% PFA. Fixed brains were cryoprotected in a sucrose gradient (15% followed by 30% overnight) and embedded in OCT compound (VWR BDH Prolab, Boxmeer, The Netherlands). Coronal cryosections (8 mm) were stained with DAPI (Invitrogen) for nuclei counterstaining. TH was stained with green fluorescent protein. Fluorescent images were captured using a laser scanning confocal microscope (LSCM, Olympus, Japan) and Volocity demo 6.2.1 software (PerkinElmer, Fremont, USA).

### 2.15. Preparation of Conditioned Medium

LCUR (5 *μ*mol) was added to hUC-MSC medium for 24 h and washed with sterile PBS three times. The cells were cultured in serum-free medium for 48 h. The supernatant was drawn and centrifuged at 4°C and 3000 r/min using an ultrafiltration tube for 1.5 h, three times, and the concentration was confirmed to be 10 times that of the original supernatant. It was then cryopreserved at -80°C. The hUC-MSC supernatant was concentrated using the same method.

### 2.16. Proliferation and Apoptosis of SH-SY5Y Detected by CCK-8 Assay and Flow Cytometry

In this study, SH-SY5Y cells treated with MPP+ (final concentration of 1000 *μ*mol/L) were used as a PD cell model. SH-SY5Y cells (5 × 10^4/^mL, 100 *μ*L) were plated in 96-well plates and divided into six groups: (1) control group: cells without treatment, (2) control+CM-MSC (CM-MSC+C) group: after SH-SY5Y cells were cultured for 12 h and 10 *μ*L of CM-MSC were added to the medium, (3) control+CM-CUR+C (CM-CUR+C): after SH-SY5Y cells were cultured for 12 h and 10 *μ*L CM-CUR were added to the medium, (4) model group: after SH-SY5Y cells were cultured for 12 h and MPP+ was added to the medium for an additional 24 h incubation, (5) model group+CM-MSC (CM-MSC+M): model group+10 *μ*L of CM-MSC, and (6) model group+CM-CUR (CM-CUR+M): model group+10 *μ*L of CM-CUR. The cells were incubated for 24 and 48 h, and 10 *μ*L of CCK8 solution was added to each well. Plates were incubated for 2 h, and absorbance was measured at 450 nm using a microplate reader.

Treated SH-SY5Y cells were digested with 0.25% trypsin and centrifuged at 1000 rpm for 10 min, and the supernatant was removed. Cells were washed twice with PBS and fixed with 70% ethanol. Cells were centrifuged at 1000 rpm for 10 min, washed in PBS twice, and adjusted to a concentration of 1 × 10^6^ cells/mL in 0.5 mL; 0.5 mL of RNase (1 mg/mL in PBS) was added to the cells. Five microliters of Annexin V-FITC was added, gently mixed, and incubated for 15 min at room temperature in the dark. After gentle mixing with propidium iodide (PI) (at a final concentration of 50 mg/L), cells were filtered and incubated in the dark at 4°C for 30 min before flow cytometry.

### 2.17. Expression of TH and MAP2 in SH-SY5Y Cells by Immunofluorescence and Western Blot

After incubation for 48 h, the supernatant was discarded. The cells were fixed in 4% PFA for 10 min. After washing in PBS twice, 5 min per time, 1% bovine serum albumin (BSA) was used for blocking for 30 min. Cells were incubated with rabbit anti-TH and MAP2 primary antibodies (rabbit IgG, 1 : 500, Abcam, Cambridge, UK) diluted in 1% BSA at 4°C overnight. Cells were rinsed with PBS twice, each time for 5 min. Cells were treated with the secondary antibody (37°C for 1 h, Abcam, Cambridge, UK). The cells were stained with 5 *μ*g/mL of DAPI (Sigma, USA) for 2 min and observed under a fluorescence microscope. After 96 h incubation with CM-CUR or CM-MSC, the cells were collected into lysis solution A and western blot was performed as described above.

### 2.18. Observation of Ultrastructure

hUC-MSCs were activated with CUR, washed with PBS three times, and centrifuged. The supernatant was discarded, and cells were transferred to 1.5 mL Eppendorf tubes and fixed in formaldehyde at 4°C for 3 days. After dehydration, embedding and staining were performed. Ultrastructural changes were observed under a transmission electron microscope.

### 2.19. Detection of Expression of Cytokines in the Supernatant by ELISA

Supernatant and coating buffer (0.5 mol/L NaHCO_3_ buffer, pH 9.6) were mixed at a ratio of 1 : 1. All ELISA kits were from IBL (Germany). All antibodies were purchased from Abcam (UK), BD Biosciences (San Jose, CA), BD (Franklin Lakes, NJ), and BioLegend (Cambridge, UK). Assays were performed according to the manufacturers' instruction.

### 2.20. Statistical Analysis

Statistical analyses were performed using SPSS 10.0 (SPSS Inc., USA). Data were presented as means ± SEM and tested using the Student *t*-test or one-way analysis of variance (ANOVA) followed by either Newman-Keuls or Bonferroni's multiple comparisons test as a post hoc test. *p* < 0.05 was considered statistically significant. In this study, all experiments were performed three times, and the mean values were used for analysis. The results of immunocytochemistry and western blotting were analyzed by an Image-Pro Plus 5.0 image analyzer (Media Cybernetics, USA). The integrated optical density (IOD) and relative abundance were evaluated by statistical analysis.

## 3. Results

### 3.1. hUC-MSC Isolation, Phenotypic Characterization, and Differentiation

**Figure 1:**
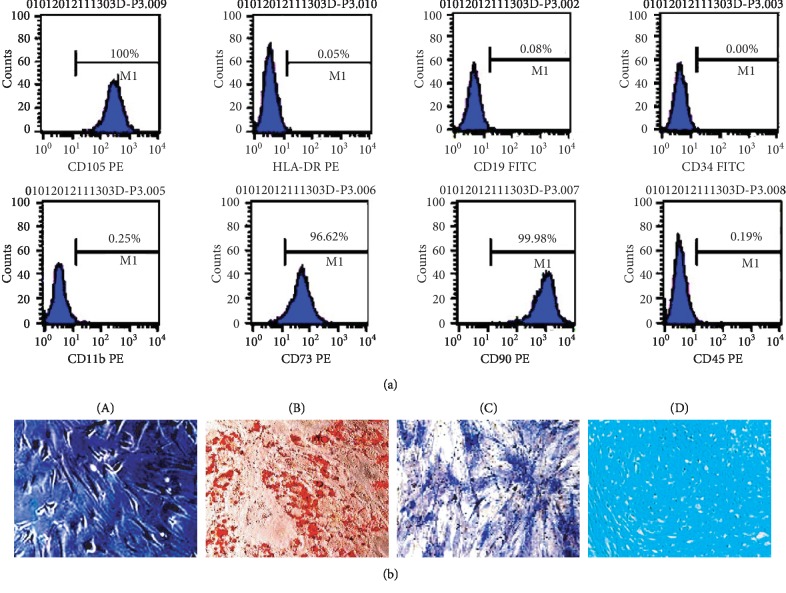
Phenotypic characterization and differentiation of hUC-MSCs. (a) Flow cytometry showing that isolated cultured hUC-MSCs highly expressed CD105, CD73, and CD90 and were negative for hematopoietic markers CD19, CD34, and CD45 and for the lymphocyte surface marker HLA-DR. (b) (A) Gross morphology of hUC-MSCs under light microscopy, (B) oil-red-O staining for the adipogenic differentiation of hUC-MSCs after 3 weeks, (C) endogenous alkaline phosphatase staining for the osteogenic differentiation of hUC-MSCs, and (D) Alcian blue staining of hUC-MSCs after 3 weeks for chondrogenic differentiation (scale bar = 100 *μ*m).

Flow cytometry showed that the hUC-MSCs were negative for the hematopoietic markers CD19, CD34, and CD45 and the lymphocyte surface markers HLA-DR, while they highly expressed the mesenchymal markers CD73, CD90, and CD105.  Figure 1(a) shows representative flow cytometry data for each marker. We next investigated their ability to differentiate into various mesenchymal lineages (Figure 1(b)), and we found that hUC-MSCs could be induced into adipose (a), bone (b), and cartilage cells (c). hUC-MSC preparations were tested for microorganisms and endotoxin before being used in animals (Table 1).

### 3.2. Preparation of hUC-MSCs-CUR

**Figure 2:**
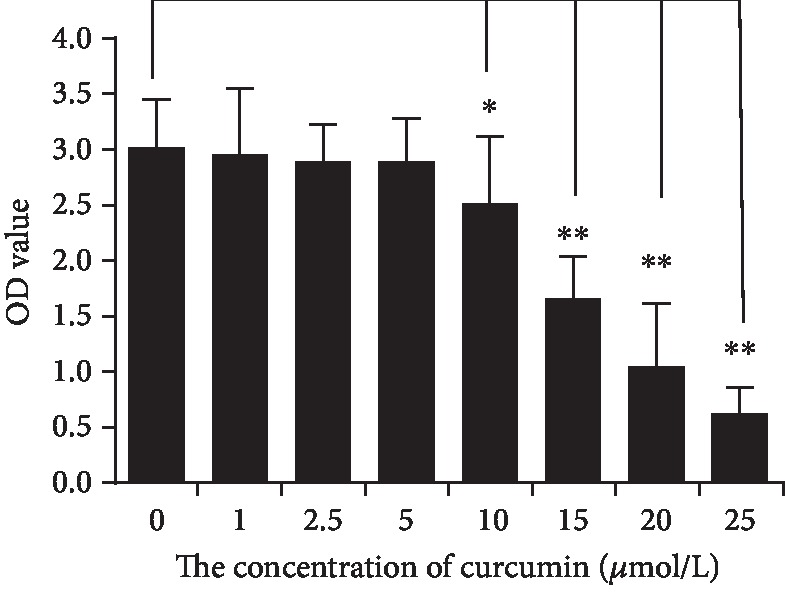
CCK-8 assay to examine the proliferation of hUC-MSCs activated with different concentrations of CUR. CUR did not stimulate proliferation and apoptosis at a concentration ranging 0-5 *μ*mol/L. When the concentration of CUR was >10 *μ*mol/L, apoptosis of hUC-MSCs occurred (^∗^*p* < 0.05 and ^∗∗^*p* < 0.01, Student's *t*-test; all experiments were performed thrice).

CCK-8 did not change with CUR in the range of 0-5 *μ*mol/L, indicating that neither proliferation nor apoptosis occurred in hUC-MSCs at these concentrations. With further increase in CUR concentration, CCK-8 of hUC-MSCs began to decrease, and a significant decrease appeared when the CUR concentration was >10 *μ*mol/L (all *p* < 0.05) (Figure 2). DMSO had no effect on CCK-8 expression (data not shown). According to these results, 5 *μ*mol/L of CUR was added to hUC-MSC medium for 24 h. The hUC-MSCs were washed with sterile PBS thrice and incubated in F12 medium with 10% serum for 48 h.

### 3.3. hUC-MSCs-CUR Transplantation Improved the Behaviors of PD Mice

**Figure 3:**
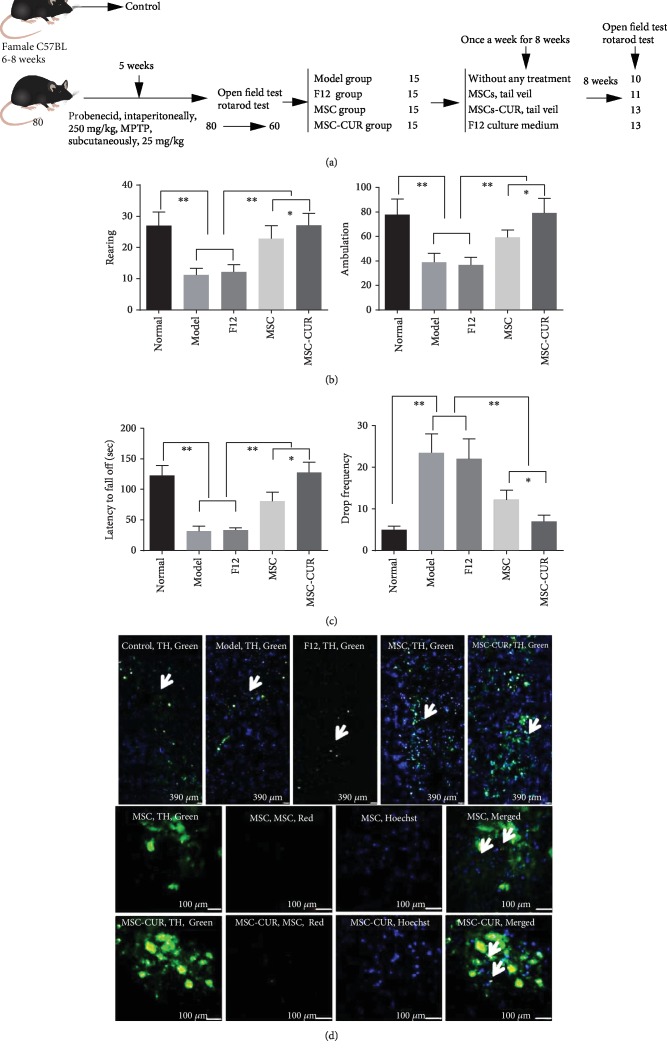
Experimental schema, behavioral changes, and MSC tracking. (a) Schema detailing method for establishment of PD mouse model and infusion MSC; the number in the box is the rest of mice. (b) Open field test. The average number of rearing in the model and F12 groups was decreased compared with that in the normal group (^∗∗^*p* < 0.01). The numbers of rearing in the MSC and MSC-CUR groups were significantly improved compared with the model group (^∗∗^*p* < 0.01). Significant difference was also found between the MSC group and the MSC-CUR group (^∗^*p* < 0.05). Changes of ambulation were consistent with that of rearing. (c) Rotarod test. The tendency of latency to fall was similar to that of rearing. Changes of drop frequency within 2 min were opposite to the changes of latency to fall. The drop frequency was increased due to limb stiffness and imbalance of movement in the model and F12 groups compared with the normal group (^∗∗^*p* < 0.01). Significant improvement was observed after stem cell treatment (^∗∗^*p* < 0.01). Moreover, the improvement was more important in the MSC-CUR group compared with the MSC group (^∗^*p* < 0.05) (one-way analysis of variance, Bonferroni's multiple comparisons test, *n* = 20, *F* = 3.252, *p* < 0.05). (d) Under LSCM, MSCs labeled with DiI (red fluorescence, shown as arrows) were presented in the striatum region of MSC and MSC-CUR group mouse brain; the change of TH expression can be clearly seen (green fluorescence).

Figure 3(a) presents a schema detailing the methods for the establishment of the PD mouse model and infusion of MSCs, with the number of animals. Compared with the control mice, the mice with PD presented typical symptoms such as unsteady gait, reduced spontaneous activities, easy irritation, straight erected tail, limb stiffness, and slow and uncoordinated limb movement. These symptoms were alleviated after treatment with hUC-MSCs and hUC-MSCs-CUR. To determine the effects of hUC-MSC and hUC-MSCs-CUR transplantation on PD mice, the open field and rotarod tests were used to examine the motor abilities.

In the open field test, the average number of rearing within 5 min in the control group was 27.6 ± 6.3, compared with 11.4 ± 3.4 in the model group (*p* < 0.01 vs. controls) and 12 ± 2.3 in the F12 group (*p* < 0.01 vs. controls). After stem cell treatment, the number of rearing was 22.8 ± 4.1 and 28.9 ± 5.4 in the MSC and MSC-CUR groups, respectively, significantly higher than in the model and F12 groups (*p* < 0.01). The difference between the MSC and MSC-CUR groups was significant (*p* < 0.05) (Figure 3(b)). Moreover, changes of ambulation were similar to those of rearing (Figure 3(b)).

As shown in Figure 3(c), the change of latency to fall was similar to that of rearing, while the general pattern of drop frequency within 2 min was opposite to that of latency to fall. Compared with controls (5 drops), the drop frequency in the model (24 drops) and F12 groups (22 drops) was increased (*p* < 0.01). The drop frequency was obviously improved in the MSC group (12 drops) and MSC-CUR group (7 drops) (*p* < 0.01). There was a significant difference between the MSC and MSC-CUR groups (*p* < 0.05).

MSCs labeled with DiI (red fluorescence) and TH protein (green fluorescence) were observed under LSCM. As shown in Figure 3(d), MSCs labeled with DiI were present in the striatum region of the brain of PD mice treated with MSC and MSC-CUR, with a change of TH expression (shown as arrows). That means that MSCs can enter into the brain of PD mice via the BBB and can differentiate into TH cells.

### 3.4. hUC-MSCs-CUR Increased TH Expression in the SN and CS

**Figure 4:**
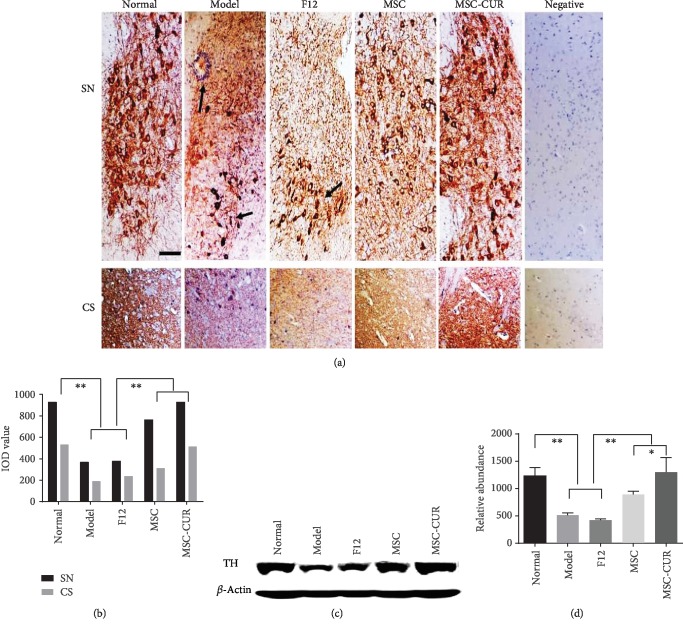
TH expressions in the CS of PD mice after cell treatment. (a, b) Immunohistochemistry for TH (10 serial CS sections of five mice from five groups (control *n* = 20, the other groups *n* = 15), taken at an interval of 4 *μ*m) showed that the numbers of TH-positive cells in the SN and CS of the model and F12 groups were greatly decreased compared with the normal mice (^∗∗^*p* < 0.01) (arrows, scale bar = 50 *μ*m). In the hUC-MSC and hUC-MSCs-CUR groups, TH-positive cells stained darker and the number of cells was obviously increased. Cell arrangement was neat, and axons became longer and denser in the SN and CS. There were significant differences compared with the model and F12 groups (^∗∗^*p* < 0.01). There was a significant difference between the MSC and MSC-CUR groups (^∗^*p* < 0.05) (one-way analysis of variance, Bonferroni's multiple comparisons test, *n* = 5, *F* = 30.21, *p* < 0.05). (c, d) Western blot showed a pattern of TH expression that was consistent with immunohistochemistry.

As shown in Figures 4(a) and 4(b), immunohistochemistry showed that the number of TH-positive cells in the SN was greatly reduced in the model and F12 groups compared with the control group. The arrangement of TH-positive cell structures was irregular; the axons and dendrites were thinner and broken, and infiltration of tangled glial cells could be observed (arrows). Brown-yellowish nerve fibers in the CS were obviously lighter. There were significant differences between controls and the model and F12 groups (*p* < 0.01).

After treatment with hUC-MSCs and hUC-MSCs-CUR, TH-positive cells stained dark and the number of cells was markedly increased. Cellular structures appeared well-organized. Axons became longer and denser. There were significant differences compared with the model group (*p* < 0.01). Moreover, significant differences in TH expression were found between the MSC and MSC-CUR groups (*p* < 0.05). More TH-positive cells with longer and darker stained axons in the CS were observed in the MSC-CUR group, compared with the MSC group. Because the SN is very small in mice, isolation from the basal ganglion is very difficult. In order to detect TH expression, proteins were extracted from whole basal ganglion for western blotting (Figure 4(c)) and the results showed a general tendency similar to that detected by immunohistochemistry (Figure 4(d)).

### 3.5. hUC-MSCs-CUR Increased the Levels of DA in the CS

**Figure 5:**
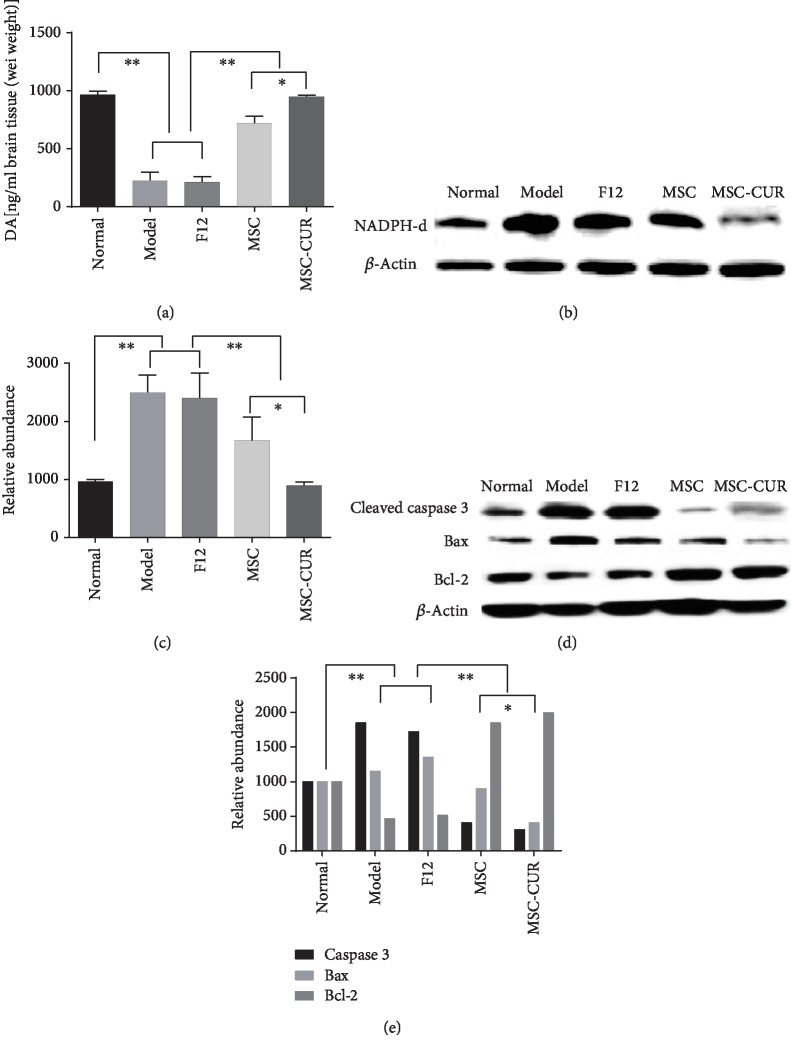
Detection of DA, NADPH-d, and apoptosis-related factors in the CS of PD mice. (a) DA levels were very low in the model and F12 groups compared with normal mice (^∗∗^*p* < 0.01), whereas the DA level was obviously increased in the hUC-MSC and hUC-MSCs-CUR groups (^∗∗^*p* < 0.01). DA concentration in the MSC-CUR group was higher than that in the MSC group (^∗^*p* < 0.05) (one-way analysis of variance, Bonferroni's multiple comparisons test, *n* = 5, *F* = 70.23, *p* < 0.05). (b, c) NADPH-d expression in the model and F12 groups was increased compared with the normal group (^∗∗^*p* < 0.01). In contrast, the levels of NADPH-d were significantly decreased in the MSC and MSC-CUR groups (^∗^*p* < 0.05; ^∗∗^*p* < 0.01). Obviously, the level of NADPH-d in the MSC-CUR group was significantly lower than that in the MSC group (^∗^*p* < 0.01). (d, e) Compared with normal mice, the model and F12 groups showed significantly reduced Bcl-2 expression in the CS, while the expression of Bax and caspase 3 was markedly upregulated (^∗∗^*p* < 0.01). In the MSC and MSC-CUR groups, Bcl-2 expression was upregulated, while the expression of Bax and caspase 3 was downregulated (^∗∗^*p* < 0.01 vs. the model and F12 groups), especially in the MSC-CUR group (^∗^*p* < 0.05, vs. the MSC group; two-way analysis of variance, Bonferroni's multiple comparisons test). All experiments were performed thrice. Protein was extracted from the brain tissues of five mice (control *n* = 20, the other groups *n* = 15, one-way analysis of variance, *n* = 5, *F* = 30.53, *p* < 0.05).

Results of high performance liquid chromatography (HPLC) revealed that compared with the control group, striatal DA levels in the model and F12 groups were significantly reduced (*p* < 0.01), while DA levels were increased after hUC-MSC and hUC-MSCs-CUR treatment (*p* < 0.01); DA levels in the MSC-CUR group were higher than those in the MSC group (*p* < 0.05) (Figure 5(a)).

### 3.6. hUC-MSCs-CUR Reduced the Levels of Nicotinamide Adenine Dinuleotide Phosphate Diaphorase (NADPH-d) in the CS

Western blot showed that NADPH-d expression in the CS was increased in the model and F12 groups compared with the control group (*p* < 0.01). In contrast, NADPH-d expression was decreased in the MSC and MSC-CUR groups (*p* < 0.05 and *p* < 0.01) (*p* < 0.01) (Figures 5(b) and 5(c)).

### 3.7. Expression of Apoptotic Factors in the CS of PD Mice

Compared with controls, expression of the antiapoptosis factor Bcl-2 in the CS was reduced in the model and F12 groups, while expression of the proapoptotic factor Bax and cleaved caspase 3 was increased (*p* < 0.01). In the MSC and MSC-CUR groups, Bcl-2 expression was upregulated and Bax and cleaved caspase 3 were downregulated (Figures 5(d) and 5(e)) (*p* < 0.01 vs. the model and F12 groups), and the effects were stronger in the MSC-CUR group compared with the MSC group (*p* < 0.05).

### 3.8. Ultrastructure of hUC-MSCs-CUR

**Figure 6:**
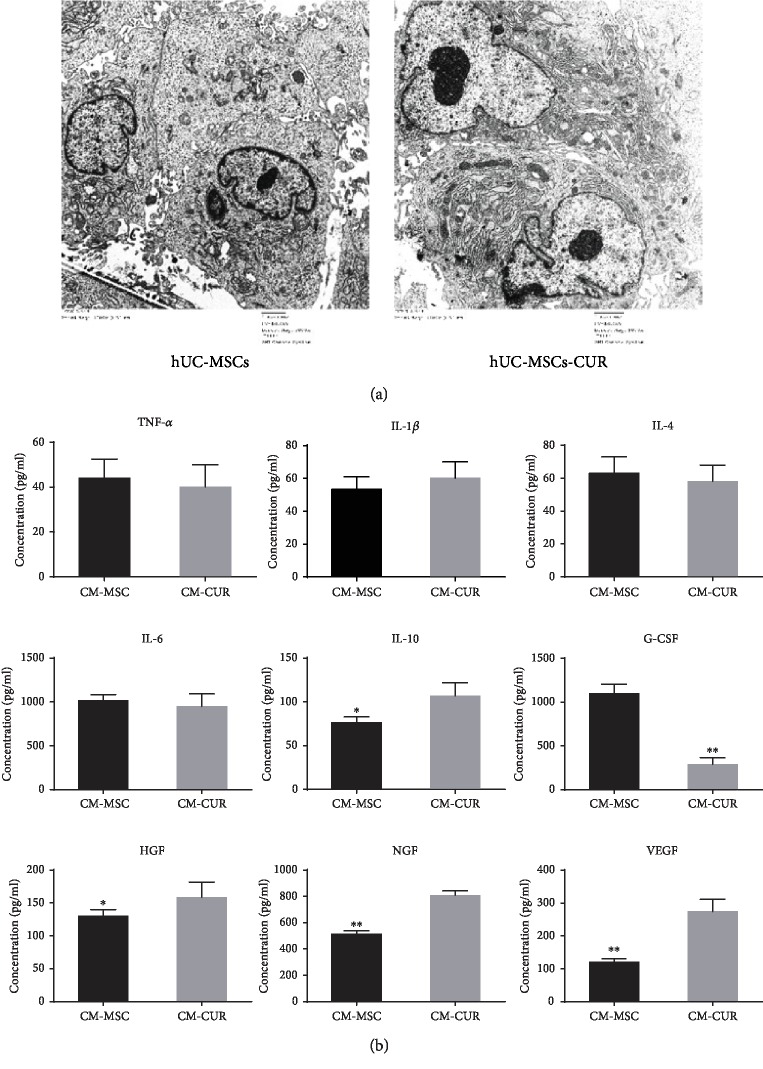
Ultrastructure of hUC-MSCs-CUR and cytokine levels in the supernatant of hUC-MSCs-CUR. (a) The number of rough endoplasmic reticulum was obviously increased in hUC-MSCs-CUR compared with hUC-MSCs (arrows, scale bar = 2 *μ*m). (b) ELISA showed that the levels of IL-10, HGF, NGF, and VEGF were increased in the supernatant of hUC-MSCs-CUR (^∗^*p* < 0.05, ^∗∗^*p* < 0.01 vs. controls). The levels of G-CSF in the supernatant of hUC-MSCs were higher than those in hUC-MSCs-CUR (^∗∗^*p* < 0.01) (Student's *t*-test). All experiments were performed thrice.

To further understand the mechanisms underlying CUR-activated hUC-MSCs, we used a transmission electronic microscope to observe the ultrastructural changes of the cells. The amount of rough endoplasmic reticulum (RER, arrows, bar = 2 *μ*m) was increased in hUC-MSCs-CUR compared with hUC-MSCs (Figure 6(a)). The RER is an important organelle in eukaryotic cells, commonly associated with the synthesis and transport of proteins. Therefore, it could be inferred that CUR apparently enhanced the secretion function of hUC-MSCs. Herein, many kinds of cytokines and growth factors in the supernatant of hUC-MSCs-CUR were detected by ELISA. Levels of IL-10, HGF, NGF, and VEGF were significantly upregulated in the hUC-MSCs-CUR group compared with the hUC-MSCs group (all *p* < 0.05). G-CSF expression was higher in the supernatant of the hUC-MSCs-CUR group compared with the hUC-MSCs group, but there were no differences for TNF-*α*, IL-1*β*, and IL-6 (Figure 6(b)).

### 3.9. CM-CUR Promoted the Proliferation of PD Model Cells

**Figure 7:**
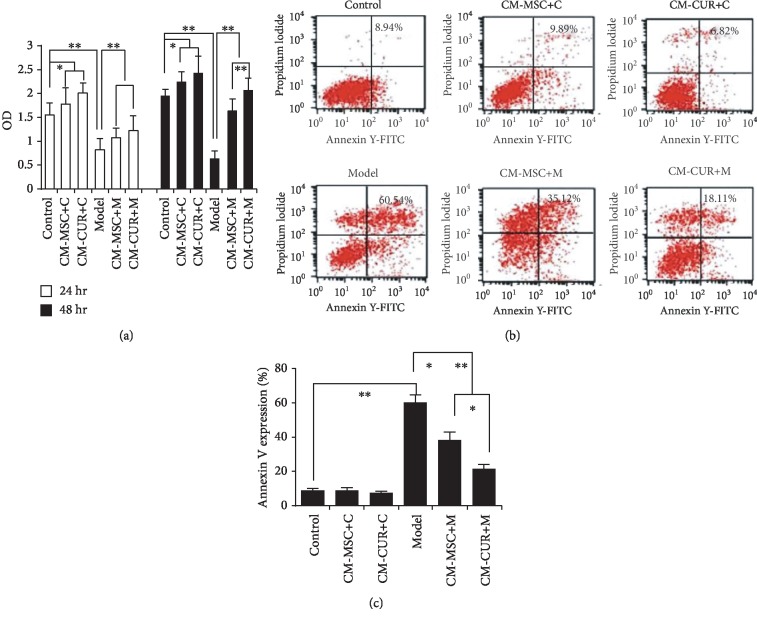
CM-CUR protect against MPTP-induced degeneration of PD model cells. (a) The proliferation of the SH-SY5Y cells in the model group was significantly declined (^∗∗^*p* < 0.01 vs. normal cells), whereas increased after treatment with CM-MSC and CM-CUR at 24 and 48 h (^∗^*p* < 0.05; ^∗∗^*p* < 0.01). At 24 h, the CM-MSC and CM-CUR groups did not display any significant difference. At 48 h, the proliferation in the CM-CUR group significantly exceeded that of the CM-MSC group (^∗∗^*p* < 0.01). (b) Flow cytometry showed that the rate of apoptosis in SH-SY5Y cells was 8.94 ± 1.2%, 60.54 ± 3.9% in the model group, 35.12 ± 4.9% in the CM-CUR group, and 18.11 ± 2.7% in the CM-MSC group. After CM-MSC and CM-CUR were added to the normal SH-SY5Y cells, the proliferation was lower, at 9.89 ± 1.3% and 6.82 ± 1.2%, respectively. (c) There were significant differences between the normal and model cells (^∗∗^*p* < 0.01). The apoptosis rate of SH-SY5Y cells in the CM-MSC and CM-CUR groups was significantly lower compared with the model group (^∗∗^*p* < 0.01), especially in the CM-CUR group. There were differences between the CM-MSC group and the CM-CUR group (^∗∗^*p* < 0.01) (two-way analysis of variance, Newman-Keuls test). All experiments were performed thrice.

After incubation with 1000 *μ*mol/L of MPP+ for 24 h, the proliferation of SH-SY5Y cells declined but was increased after treatment with CM-MSC and CM-CUR for 24 and 48 h (*p* < 0.05 and *p* < 0.01). At 24 h, the CM-MSC and CM-CUR groups did not display any significant difference. At 48 h, the proliferation in the CM-CUR group significantly exceeded that of the CM-MSC group (*p* < 0.01) (Figure 7(a)).

Apoptosis was examined using Annexin V/PI double staining. The apoptosis rate of the SH-SY5Y cells was detected by flow cytometry at 48 h. Results showed that the apoptosis rate was 8.94 ± 1.2% in normal SH-SY5Y cells, 9.89 ± 1.3% in the CM-MSC+C group, and 6.82 ± 1.2% in the CM-CUR+C group, compared with 60.54 ± 3.9% in the model group, 35.12 ± 4.9% in the CM-MSC+M group, and 18.11 ± 2.7% in the CM-CUR+M group (Figures 7(b) and 7(c)). There were significant differences between the normal and model cells (*p* < 0.01). Under treatment with CM-MSC and CM-CUR, the apoptosis rate of SH-SY5Y cells significantly declined (*p* < 0.01), especially in the CM-CUR group and there were differences between the CM-MSC and CM-CUR groups (*p* < 0.01) (Figure 7(c)).

### 3.10. CM-CUR and CM-MSC Increased the Expression of TH and MAP2

**Figure 8:**
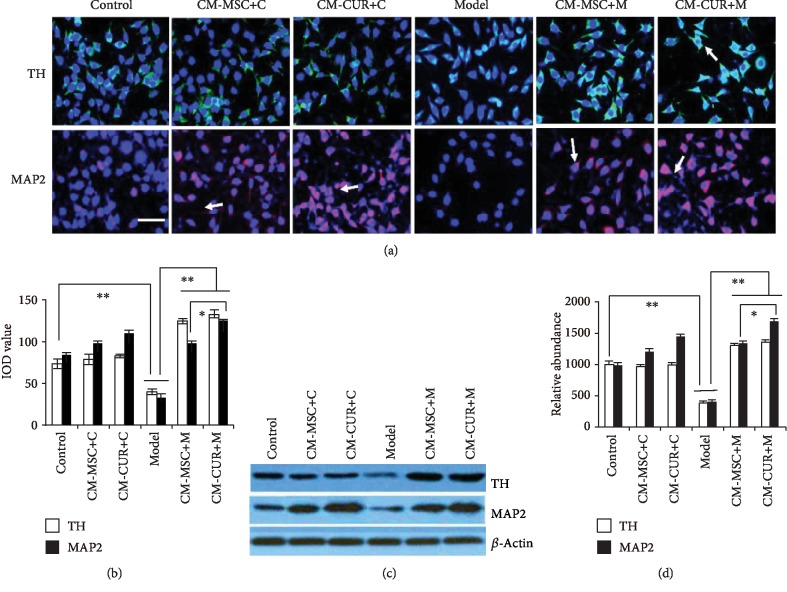
Detection of TH and MAP2 in SH-SY5Y cells treated with CM-CUR and CM-MSC by immunofluorescence and western blot. (a) TH and MAP2 expression was upregulated in SH-SY5Y cells treated with CM-MSC and CM-CUR. Obvious morphological changes were observed in SH-SY5Y cells. The cells had many long processes as long as more than 2-fold that of the cell. These processes were similar in morphology to axons (arrows, scale bar = 50 *μ*m). These morphological changes were remarkable in the CM-CUR group. (b) Image and statistical analyses of (a). TH and MAP2 expression in the normal control was stronger than that in the model group (^∗∗^*p* < 0.01). TH and MAP2 expression was increased after treatment with CM-MSC and CM-CUR (^∗∗^*p* < 0.01). Significant difference in upregulation of MAP2 expression was observed between the CM-MSC and CM-CUR groups (^∗^*p* < 0.05). (c, d) The expression of TH and MAP2 was detected by western blot. The pattern was similar to that detected by immunofluorescence (one-way analysis of variance, Bonferroni's multiple comparisons test, *n* = 3, *F* = 110.65, *p* < 0.05). All experiments were performed thrice.

Expression of TH and MAP2 in the SH-SY5Y cells declined due to MPP+ injury compared with normal cells (*p* < 0.01). After 48 h of treatment with CM-MSC and CM-CUR, immunofluorescence and western blot showed significant upregulation of TH and MAP2 in the CM-MSC+M and CM-CUR+M groups (*p* < 0.01 vs. the model group). Significant difference in MAP2 expression was found between the CM-MSC and CM-CUR groups (*p* < 0.05) (Figure 8). TH and MAP2 were upregulated in SH-SY5Y cells treated with CM-CUR and CM-MSC, suggesting that SH-SY5Y cells gradually differentiated into DA neurons.

There were morphological changes of SH-SY5Y cells. Normal SH-SY5Y cells had a spindle shape similar that of fibroblasts; they usually had 2-3 processes and were relatively short. On the other hand, the edge of SH-SY5Y cells treated with supernatant became dim and with many long processes with a length of more than two cell body lengths. The morphology of these processes was similar to that of axons (as indicated by arrows). This phenomenon was most evident in the CM-CUR group (Figure 8).

After normal SH-SY5Y cells were treated with CM-MSC and CM-CUR, TH expression did not change in the CM-MSC+C and CM-CUR+C groups, while MAP2 expression was increased. Cells in the CM-MSC+M group and the CM-CUR+M group had strong MAP2 expression and morphological changes, but no significant difference between the CM-MSC+M and CM-CUR+M groups was observed (Figure 8). These results suggested that CM-MSC and CM-CUR could promote the differentiation of normal cells to some extent.

## 4. Discussion

Our research group has been studying CUR for years, particularly the effects of CUR for the treatment of Alzheimer disease (AD) [34, 35]. In recent years, in regard to similarities between AD and PD as degenerative diseases of the central nervous system, we hypothesized that CUR could also be used for the treatment of PD. In our previous work, we found that curcumin was very difficult to be absorbed in the abdominal cavity of mice, due to its extremely low bioavailability. Therefore, we speculated whether combination of hUC-MSCs and CUR could have a better effect as PD treatment. In the preliminary experiments, we found that 5 *μ*mol/L of CUR did not have any treatment effect, while antioxidation and antiapoptotic effects were shown at 25 *μ*mol/L of CUR in PD mice, which was consistent with the literature [24]. Nevertheless, in cell experiments, few cells survived to 25 *μ*mol/L of CUR. Therefore, the therapeutic concentrations of CUR are not the same in cell and animal experiment and comparisons cannot be made between the two models. Therefore, there was no CUR treatment group and we focused on examining the effects of hUC-MSCs and CUR-activated hUC-MSCs for the treatment of PD.

First, we examined the effects of CUR-activated hUC-MSCs on a PD animal model induced by MPTP/probenecid, which is the most common animal model of PD [36, 37]. We found that hUC-MSCs-CUR transplantation significantly improved the motor abilities of PD mice, such as limb stiffness and slow and uncoordinated limb movement. In addition, the numbers of DA neurons and TH expression were higher in the MSC-CUR group than that in the MSC group.

We also studied the oxidative stress level in the PD mice with and without cell treatment. Although the pathogenesis of PD is still poorly understood, it is believed that oxidative stress by oxygen free radicals plays an important role. It is known that there are at least three isoforms of nitric oxide synthase (NOS): inducible (iNOS), endothelial (eNOS), and neuronal (nNOS). In the process of NO synthesis, NADPH-d is required as a coenzyme for electron transport. Many authors consider that NADPH-d levels in the nervous system can be used as a surrogate for NOS expression [38]. In the present study, NADPH-d expression in the striatum was markedly decreased after cell treatment, suggesting that NOS expression was decreased, particularly in CUR-activated hUC-MSCs. With the downregulation of NOS expression, CUR-activated hUC-MSCs had a better resistance to oxidative stress, which could promote the survival of the DA neurons. Detection of the apoptosis factors revealed that the antiapoptotic factor Bcl-2 was significantly upregulated in the MSC-CUR group compared with the MSC group, while the expression of the proapoptosis factors Bax and cleaved caspase 3 was markedly decreased. Therefore, these results strongly suggest that hUC-MSCs-CUR had strong differentiation and repair potentials, as well as antioxidation and antiapoptosis abilities. Such results are in line with our hypothesis based on our previous work in AD [34, 35], i.e., CUR and hUC-MSC could be used for PD treatment.

Subsequently, experiments were performed to observe the protective effects of hUC-MSCs-CUR supernatant on the SH-SY5Y cellular model of PD. The results of the CCK-8 assay and flow cytometry revealed that hUC-MSCs-CUR supernatant significantly promoted the proliferation of PD model cells. Obviously, this effect was better than that of hUC-MSCs without CUR.

In addition, using immunofluorescence, we found that SH-SY5Y cells became dim and with many long processes with a morphology similar to axons when exposed to hUC-MSC and hUC-MSCs-CUR supernatant for 48 h. This phenomenon was more evident in the CM-CUR group. Western blot results confirmed the expression of MAP2 in the CM-CUR group. MAP2 is a neuron-specific protein that stabilizes microtubules in the dendrites of postmitotic neurons. At the same time, the levels of TH in the CM-MSC and CM-CUR group were also increased. TH is the key enzyme for the biosynthesis of DA and is considered as a marker of DA neurons [11]. Therefore, immunofluorescence revealed that CM-CUR could promote the SH-SY5Y cellular model of PD to differentiate into DA neurons in some extent. These data perhaps supported the animal model results and strongly suggest that CUR-activated hUC-MSCs could be used to treat PD.

To further examine the mechanisms underlying the treatment effect of CUR-activated hUC-MSCs, we observed the ultrastructural changes of CUR-activated hUC-MSCs. We found that RER in the CUR-activated hUC-MSCs were more abundant than those in the hUC-MSCs. RER are complex functional structures made of well-arranged flat sac-like ribosomes and endoplasmic reticulum and connect with the outer membrane of the nucleus. The main function of RER is the synthesis of large protein molecules [39]. Therefore, cells with abundant protein synthesis usually have relatively well-developed RER [40]. ELISA showed that the expression of IL-10, HGF, NGF, and VEGF was upregulated in the supernatant of hUC-MSCs-CUR compared with hUC-MSCs. Only G-CSF expression was increased in the hUC-MSC supernatant. Recent studies showed that immune reactions and inflammation are involved in the pathogenesis of PD, and attention has been given to the roles of cytokines in the progression and treatment of PD [41–44]. IL-10 can alleviate inflammatory reactions and inhibit the production and release of many proinflammatory factors such as TNF-*α*, IL-1*β*, and IL-6 [41–43]. IL-4 is also an anti-inflammatory factor, but no significant change of IL-4 was observed. HGF is a multifunctional growth factor involved in the physiopathology of PD [45–47]. VEGF is a vascular growth factor induced by hypoxia; it is expressed abundantly in the brain. It has extensive biological effects such as neurotrophy, neuroprotection, antiapoptosis, and cell proliferation and enhances the osmolarity of the capillaries. Recent research has shown that VEGF is associated with many central nervous system diseases such as ischemic diseases, AD, and PD [48]. NGF is an important neurotrophic factor that plays a significant role in PD treatment [49]. Pretreatment with G-CSF can protect DA neurons against neurotoxicity induced by 6-OHDA [50–52]. However, our results are in contradiction with these reports, i.e., hUC-MSCs-CUR generated a relatively small amount of G-CSF. In conclusion, compared with hUC-MSCs, transplantation of hUC-MSCs-CUR could improve the motor recovery of PD. These beneficial effects could be associated with the combination of the effects of hUC-MSCs and CUR.

In recent years, many authors provided new perspectives for the application of MSCs to PD therapy. Wang et al. suggest that hypoxia can promote MSC proliferation and DA neuronal differentiation and can be used for intrastriatal transplantation [32]. Teixeira et al. tried to inject MSC secretome into the SN and CS. The results showed that the secretome potentiated the increase of DA neurons and neuronal terminals in the SNC and STR. Mass spectrometry analysis and Bio-Plex assays revealed that the important neuroregulatory molecules in MSC secretome were mainly cystatin C, glia-derived nexin, galectin-1, pigment epithelium-derived factor, vascular endothelial growth factor, brain-derived neurotrophic factor, interleukin-6, and glial cell line-derived neurotrophic factor [53]. In a study by Yao et al., NSCs were cultured using lyophilized MSC culture medium and transplanted into PD rat models. The results suggest that the NSCs possess a great potential as a graft candidate for the treatment of PD [54]. Interestingly, according to Leveque, the rats transplanted with both porcine neuroblasts (pNb) and MSC exhibited healthy porcine neurons in the striata and displayed healthy grafts with pNF70+ and TH+ neurons at 120 days posttransplantation, while no porcine neurons were detected in the rats that received only pNb at day 63 [55].

## 5. Conclusion

We still have a lot of work to do in the future before using MSCs for the treatment of PD. Inspired by excellent previous studies, future steps will be to detect the important neuroregulatory molecules of MSC-CUR using mass spectrometry analysis and Bio-Plex assays, to compare the effects of different transplantation methods of MSC-CUR and to explore which cells MSC-CUR can be combined with. Additional studies are necessary before clinical trials can be carried out.

## Figures and Tables

**Table 1:** Safety tests of human umbilical cord-derived mesenchymal stem cells (hUC-MSCs).

Microbiological and other detection	Results
Aerobic bacteria	Negative
Anaerobic bacteria	Negative
Fungus	Negative
Hepatitis B virus surface antigen (HBsAg)	Negative
Hepatitis B virus core antigen (HBcAb)	Negative
Hepatitis C virus antibodies (HCV-Ab)	Negative
Antihuman immunodeficiency virus (HIV (I+II))	Negative
Cytomegalovirus (CMV)—IgM	Negative
Human T-lymphotropic virus HTLV-I/II	Negative
Anti-Epstein-Barr virus (EBV) Ab	Negative
Syphilis	Negative
Endotoxin	1.0 EU/mL
Alanine transaminase (ALT)	4 U/L
Residual growth factor	0.1 ng/mL

## Data Availability

The data used to support the findings of this study are available from the corresponding authors upon request.
